# Manejo complejo del hematoma intramural de aorta descendente: a propósito de un caso

**DOI:** 10.47487/apcyccv.v1i3.75

**Published:** 2020-09-30

**Authors:** Juan Manuel Torres-Restrepo, Cristhian Felipe Ramírez-Ramos, Héctor Conrado Jiménez-Sánchez, Edwin Romero, Mario Espinosa-Moreno, Clara Saldarriaga

**Affiliations:** 1 Departamento de Cirugía General, Hospital Universitario Hernando Moncaleano Perdomo. Neiva, Colombia.; 2 Departamento de Cardiología Clínica, Universidad Pontificia Bolivariana y Clínica CardioVID. Medellín, Colombia. Universidad Pontificia Bolivariana Departamento de Cardiología Clínica Universidad Pontificia Bolivariana y Clínica CardioVID Medellín Colombia; 3 Departamento de Cirugía General, Universidad del Bosque. Bogotá, Colombia. Universidad El Bosque Departamento de Cirugía General Universidad del Bosque Bogotá Colombia; 4 Departamento de Cirugía Vascular Periférico, Hospital Universitario Hernando Moncaleano Perdomo. Neiva, Colombia. Departamento de Cirugía Vascular Periférico Hospital Universitario Hernando Moncaleano Perdomo Neiva Colombia; 5 Departamento de Cardiología Clínica e Insuficiencia Cardiaca, Universidad Pontificia Bolivariana, Universidad de Antioquia y Clínica CardioVID. Medellín, Colombia. Universidad de Antioquia Departamento de Cardiología Clínica e Insuficiencia Cardiaca Universidad Pontificia Bolivariana, Universidad de Antioquia y Clínica CardioVID Medellín Colombia

**Keywords:** Enfermedades de la aorta, Procedimientos Endovasculares, Hematoma, Aortic Diseases, Endovascular Procedures, Hematoma

## Abstract

Los síndromes aórticos agudos (SAA) incluyen una variedad de enfermedades anatómicas y clínicas que se superponen. El hematoma intramural (HIM), la úlcera aórtica penetrante y la disección aórtica ocurren de manera aislada o pueden coexistir en un mismo paciente. El HIM representa el 5-30% de todos los SAA y el 60-70% de los casos se localizan en la aorta descendente. El diagnóstico recae en un alto índice de sospecha clínica y en el empleo de imágenes complementarias. El manejo es conservador, pero los pacientes con algunas características de alto riesgo tienen mayor probabilidad de muerte en la fase aguda por lo que debe considerarse inicialmente el manejo endovascular. Se presenta el caso de un paciente de 69 años a quien se diagnosticó un HIM en el curso de una emergencia hipertensiva y que requirió manejo híbrido por las características anatómicas de alto riesgo para manejo endovascular aislado.

## Abstract (en)

Acute aortic syndromes (AAS) include a variety of overlapping anatomical and clinical conditions. Intramural hematoma (IMH), penetrating aortic ulcer, and aortic dissection occur in isolation or may coexist in the same patient. IMH represents 5-30% of all AAS and 60-70% of cases are located in the descending aorta. The diagnosis relies on a high index of clinical suspicion and on the use of complementary images. Management is conservative, but patients with some high-risk characteristics have a higher risk of mortality in the acute phase, so initial endovascular management should be considered. We present the case of a 69-year-old patient, in whom IMH was diagnosed in the course of a hypertensive emergency and who required hybrid management due to high-risk anatomical characteristics for endovascular management only.

El síndrome aórtico agudo (SAA) es un proceso agudo en la pared aórtica, causado por la disrupción de la capa media en un grado variable y con riesgo de ruptura de la aorta así como de otras complicaciones ^1^. El hematoma intramural (HIM) junto con la disección aórtica son entidades incluidas en los SAA; mientras que la disección aórtica clásica ocurre por una ruptura inicial en la íntima con propagación del flujo sanguíneo en un falso lumen, en el HIM la hemorragia ocurre dentro de la pared del vaso en ausencia de daño primario en la íntima ^2^. La prevalencia del HIM oscila entre 5 a 27% de todos los casos de SAA siendo el mayor compromiso en la aorta descendente ^3^, estos pueden extenderse al arco aórtico o la aorta ascendente, situación denominada HIM retrógrado tipo A, que conlleva mayor riesgo de complicaciones cardiacas y neurológicas, aquí la cirugía abierta o una aproximación híbrida se proponen como la estrategia de manejo ^4^.

Se presenta el caso de un paciente con dolor torácico cuya etiología fue un HIM desde la emergencia de la arteria subclavia izquierda. El paciente persistió sintomático pese al manejo y, por las características anatómicas de alto riesgo, se realizó un manejo híbrido exitoso. 

## Caso clínico

Paciente masculino de 69 años, sin antecedentes importantes, quien consulta al servicio de urgencias por cuadro de seis horas de evolución de dolor torácico opresivo, irradiado a dorso, acompañado de disnea y diaforesis, sin otra sintomatología. A su ingreso, el paciente estaba sintomático, sin signos de inestabilidad hemodinámica, con cifras tensionales elevadas (tensión arterial 200/110 mmHg) y sin otras alteraciones, por lo que se inicia manejo antihipertensivo y es trasladado a unidad de cuidados intensivos.

**Figura 1:**
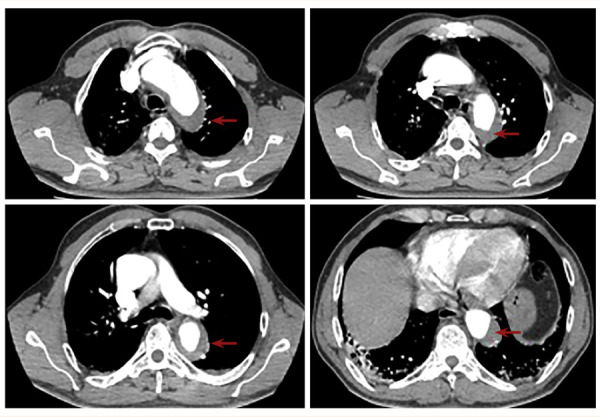
Angiotomografía de aorta torácica, cortes axiales. Se aprecia hematoma intramural que inicia desde la emergencia de arteria subclavia izquierda hasta hiato aórtico, con diámetro mayor a 20 mm. Diámetro mayor de 5 cm en aorta descendente

**Figura 2:**
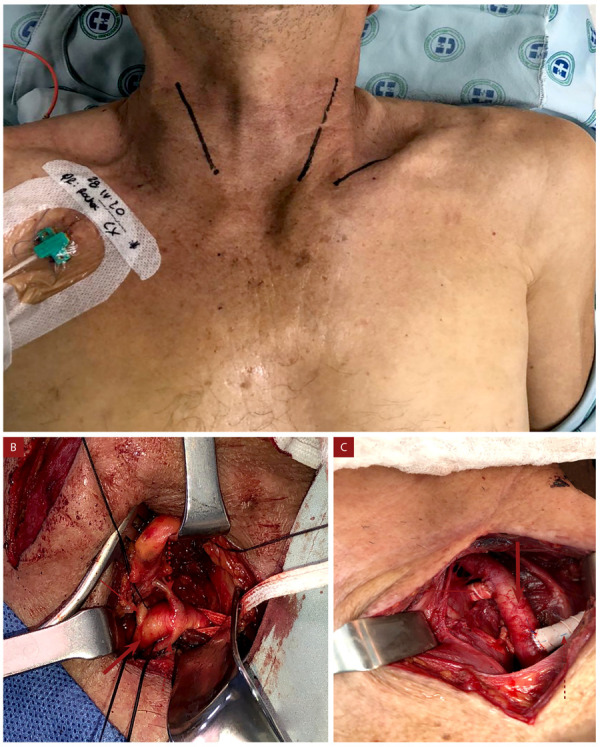
A) Cervicotomía lateral bilateral, y posterior realización de *shunts* arteriales de con injerto anillado de 7 mm carotideo-carotideo retroesofágico. B) Exposición supraclavicular muestra: arteria subclavia izquierda (flecha gruesa), arteria vertebral izquierda (flecha delgada), tronco tirocervical (flecha gruesa discontinua) y arteria torácica interna (flecha delgada discontinua). C) *Bypass* carotideo-subclavio muestra arteria carótida común izquierda (flecha gruesa), *bypass* PTF carotideocarotideo (flecha delgada) y carotideo-subclavio (flecha discontinua).

El electrocardiograma inicial no mostró cambios agudos de lesión, necrosis o isquemia, con troponina de alta sensibilidad no detectable por lo que se descartó un síndrome coronario agudo. Persistió con dolor torácico y, ante las características de este, se consideró descartar un SAA por lo que se realizó una angiotomografía de aorta torácica en la que se documentó un HIM desde la emergencia de la arteria subclavia izquierda hasta el hiato aórtico con un diámetro mayor a 20 mm **(**Figura 1**)**. Pese a lograr control de las cifras tensionales y de la frecuencia cardiaca, el paciente persistió sintomático por lo que se propuso realizar manejo quirúrgico. Las características anatómicas de la lesión eran de alto riesgo (compromiso de la emergencia de arteria carótida izquierda en íntima relación a la emergencia de subclavia) planteando la imposibilidad de fijar la endoprótesis con riesgo de lesión por oclusión a nivel de la circulación de la arteria carótida y subclavia. Por lo anterior, se realizó un manejo híbrido: en el primer tiempo quirúrgico se efectuó una cervicotomía lateral **(**Figura 2A**)** y con el uso de *shunts* arteriales se realizó un *bypass* con injerto anillado de 7 mm carotideo - carotideo retroesofágico **(**Figura 2B**)** y carotideo - subclavio izquierdo (tercio medio) **(**Figura 2C**)**. 

**Figura 3:**
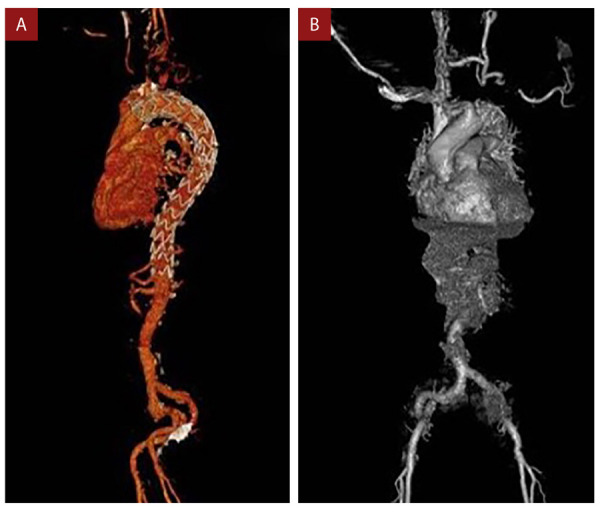
Angiotomografía de aorta toracoabdominal. Reconstrucciones donde se aprecia la endoprótesis, sin endofugas **(A)** ni aumento del hematoma **(B)**

Luego del procedimiento presentó derrame pleural izquierdo con incremento paulatino; a las 72 horas de la cirugía mostró inestabilidad hemodinámica por lo que se le trasladó a la unidad de cuidado intensivo para soporte ventilatorio y vasoactivo. En una angiotomografía de control se observó imágenes sugestivas de progresión del hematoma y signos de disección aórtica a este nivel, sin compromiso de aorta abdominal, ni de sus ramas. Posteriormente se realizó un implante de prótesis de aorta torácica descendente por vía endovascular. El procedimiento transcurrió sin complicaciones, sin presentar deterioro neurológico, con perfusión cerebral y de miembro superior izquierdo preservada. Las imágenes de seguimiento mostraron adecuada posición de la prótesis sin fugas del medio de contraste por lo que se dio alta hospitalaria 20 días después del ingreso para continuar el seguimiento **(**Figura 3**)**. 

## Discusión

Se presenta el caso de un paciente con un HIM sintomático pese al manejo médico, con necesidad de intervención quirúrgica que se realizó de manera exitosa por técnica hibrida debido a las características anatómicas del paciente.

Los síndromes aórticos agudos son emergencias vasculares que se asocian con importante morbimortalidad a corto y largo plazo ^5^. En este grupo se incluyen el HIM, la úlcera penetrante (UAP) y la disección aórtica aguda (DAA). La incidencia de esta enfermedad se estima en 2,6 a 3,5 casos por 100 000 personas año, la gran mayoría de los cuales son DAA (65-75%), seguidos por HIM (4-32%) y UAP (<10%), teniendo presente que estas lesiones pueden coexistir ^6^. Por largo tiempo se ha considerado que los HIM resultan de la ruptura espontánea del *vasa-vasorum* en la capa media del vaso, por estrés, fragilidad, hipertensión o inflamación con ausencia de una clara comunicación entre el lumen y la pared ^6^; sin embargo, con la mejoría de las imágenes se pueden detectar pequeños desgarros intimales en el 70-80% de los casos, quizás representando el sitio de lesión inicial ^6^; estas disrupciones intimales focales (DIF) cuando son menores de 3 mm se asocian con resultados favorables ^7^.

En general, la presentación clínica es similar en todos los grupos de la enfermedad, pero los pacientes con HIM, típicamente, son mayores comparados con la DA (69 vs. 62 años); existe preponderancia de hombres, con mayor incidencia de hipertensión y ateroesclerosis ^6^. El 90% de los casos son espontáneos, pero pueden relacionarse con trauma o intervenciones por catéter ^6^. Los pacientes pueden presentar signos y síntomas relacionados con complicaciones vasculares ^8^. Más del 90% presenta dolor torácico súbito, transfixiante, y muy agudo, que se puede referir al cuello o el dorso; este dolor es común tanto en los HIM tipo A como en los tipo B y puede persistir después del manejo ^8^. Típicamente, los pacientes están taquicárdicos e hipertensos y la hipotensión, cuando está presente, es un signo que sugiere complicaciones ^8^. La oclusión de las ramas, la presión sanguínea diferencial, los déficits de pulsos y la disfunción de la válvula aórtica son menos comunes en el HIM ^9^.

El diagnóstico del HIM es un reto clínico, incluso por imágenes. La tomografía computarizada y la resonancia nuclear magnética son modalidades tridimensionales con capacidad de realizar imágenes de toda la extensión del vaso y con mayor sensibilidad y especificidad comparado con el ecocardiograma transesofágico (90-100% vs. 86-75% respectivamente) ^6^. El manejo del HMI está encaminado a reducir el riesgo de complicaciones, por tal razón es necesario el control óptimo de la frecuencia cardiaca (FC<60x min), tensión arterial sistólica (TAS entre 100 - 120 mmHg), control analgésico, reposo absoluto y el seguimiento en unidad de cuidados intensivos ^6^.

En aquellos pacientes con HIM de la porción descendente de la aorta la mortalidad intrahospitalaria es menor del 10%, la literatura recomienda el manejo médico como aproximación inicial en este grupo de personas ^10^; sin embargo, los pacientes con persistencia de síntomas, pese al manejo médico, y/o que presentan inestabilidad hemodinámica, signos de ruptura aórtica, disrupciones intimales focales (con orificio comunicante >3 mm), un diámetro máximo aórtico >55 mm y crecimiento del diámetro aórtico de manera rápida durante la estancia hospitalaria, tienen indicación de manejo quirúrgico, por ser características de alto riesgo de mortalidad en la fase aguda. La recomendación inicial es un abordaje endovascular ^5^ y, como alternativa, la cirugía abierta según las características anatómicas, las comorbilidades del paciente, las restricciones propias a la endoprótesis, así como la experiencia de los grupos de atención ^1^. En años previos, la cirugía abierta era la estrategia de elección, pero debido a su alta mortalidad (25 - 50%) y morbilidad, la estrategia actual es el procedimiento menos invasivo y con mejor perfil de seguridad ^11^.

La reparación endovascular de aorta torácica (TEVAR) es un procedimiento que ha reducido la mortalidad (1,9%), y logrado una menor frecuencia de lesión neurológica con disminución en la estancia en UCI y en hospitalización ^(^^12^, por lo que es el método de manejo inicial. Para el manejo endovascular es necesario cumplir un prerrequisito esencial (sitio de anclaje proximal de la endoprótesis de mínimo 2 cm sin compromiso por la lesión), el cual no se cumplía en el paciente, ya que el hematoma y la posterior disección comprometían la emergencia de la carótida y subclavia izquierda. Se han mencionado diversas técnicas para el manejo de la oclusión de los vasos cervicales izquierdos (oclusión de la arteria subclavia sin revascularización; puente extraanatómico, puente carotideo-carotideo, transposición de arteria subclavia, puente carotideo-subclavio) ^13^; sin embargo, trabajos recientes concluyen que no hay diferencia en cuanto a la morbimortalidad, endofuga o cambios en el diámetro del saco de pacientes a los cuales se les ocluye o se les revasculariza la arteria subclavia izquierda ^14^^,^^15^. En el presente caso, debido al compromiso de ambas ramas cervicales izquierdas (carótida y subclavia), y con el fin de lograr un adecuado sitio de anclaje de la endoprótesis, se optó por una estrategia quirúrgica híbrida en dos tiempos desarrollados sin complicaciones.

## Conclusiones

El manejo quirúrgico del HIM ha cambiado con el advenimiento de la terapia endovascular, que ha demostrado una disminución en la morbimortalidad asociada con estos procedimientos, y con buenos resultados posquirúrgicos a corto, mediano y largo plazo, aun más en grupos de alto riesgo en los que las condiciones del paciente suponen un reto en la intervención, como lo es el compromiso de las ramas cervicales de la aorta. 
